# Uterine septum and reproductive outcome. From diagnosis to treatment. How, why, when?

**DOI:** 10.52054/FVVO.14.1.002

**Published:** 2022-04-03

**Authors:** A Daniilidis, P Papandreou, G.F. Grimbizis

**Affiliations:** 2^nd^ University Department in Obstetrics and Gynecology, Hippokratio General Hospital, School of Medicine Aristotle University of Thessaloniki, Greece; Gynae-oncology Department, Maidstone Hospital, MTW Trust, Kent, UK; 1^st^ University Department in Obstetrics and Gynecology, Papageorgiou Hospital, School of Medicine Aristotle University of Thessaloniki, Greece.

**Keywords:** Septate uterus, diagnosis, pathophysiology, prevalence, reproductive outcome, metroplasty

## Abstract

**Background:**

Septate uterus is a benign congenital malformation and represents the most common uterine anomaly in women with poor reproductive outcome.

**Objectives:**

To review the available scientific data concerning the biological context of the intrauterine septum and the association with poor reproductive outcome, the best methods for diagnosis and treatment.

**Materials and methods:**

From July 2020 to October 2020, we searched for relevant free full text articles in PubMed, written in English, and published from the 1st of January 2000 to 31st of July 2020.

**Main outcome measures:**

Association of the pathophysiology of septate uterus with poor reproductive outcome, evaluation of the different classification systems, the accuracy of diagnostic methods and the efficacy of the available treatment options.

**Results:**

259 articles were screened, and 22 articles were finally included in our study. Many theories regarding the pathophysiology of this congenital anomaly and its’ association with reproductive problems have been proposed along the recent decades. Combination of diagnostic methods should be used to avoid misclassification of this congenital anomaly.

**Conclusions:**

Lack of uniformity in the different classification systems makes the diagnosis of septate uterus challenging as there is no universally accepted definition. Data regarding the reproductive outcome of women with septate uterus are still limited, thus recommendations regarding optimal treatment of these women are biased.

**What is new?:**

According to new insights regarding the pathophysiology of the uterine septum, differences in the underlying embryological defects are associated with changes in the histological composition and vascularisation of septa, as well as in clinical significance.

## Introduction

Septate uterus is a congenital malformation of the female genital tract. It is defined as a deviation from normal anatomy resulting from embryological maldevelopment of the Müllerian duct which leads to the presence of a midline septum into the uterine cavity (Grimbizis et al., 2013). The embryological defects in the fusion of the Müllerian ducts, and the absorption of their connecting tissue, which seem to be responsible for septate uterus, take place prior to the 20th embryonic week (American Society for Reproductive Medicine, 2016). Septate uterus is a benign condition and represents the most common uterine anomaly in women with poor reproductive outcomes (Salim et al., 2003, Rikken et al., 2019). The intrauterine septum may divide the uterine cavity partly, above the level of the internal cervical os, or completely, up to the level of the internal cervical os (Grimbizis et al., 2013). It has a prevalence of 2-3% in women of reproductive age and is associated with infertility and other reproductive problems. Septate uterus is also associated with a higher risk of perinatal complications and lower clinical pregnancy rates (Rikken et al., 2019). Although septate uterus seems to affect the reproductive outcome in women of reproductive age, many women do not experience any reproductive difficulties (American Society for Reproductive Medicine, 2016; Rikken et al., 2019). Many criteria have been proposed for the definition of septate uterus, however, the following three are the main ones in current use: the American Fertility Society’s (AFS) currently American Society of Reproductive Medicine (ASRM) criteria, the Congenital Uterine Malformation by Experts (CUME) criteria and the European Society of Human Reproduction and Embryology (ESHRE) and European Society for Gynaecological Endoscopy (ESGE) consensus on the classification of female genital tract congenital anomalies criteria (Grimbizis et al.,2013; ASRM, 2016; Ludwin et al., 2018). Unfortunately, there is still no universally accepted definition of the septate uterus. Diagnosis of septate uterus includes radiological methods such as hysterosalpingography (HSG), magnetic resonance imaging (MRI) and three- dimensional (3D) sonography and invasive methods such as hysteroscopy and laparoscopy that offer direct visualisation of the interior and the exterior of the uterus. Still, none of the above methods is considered the gold standard in the recent era. Treatment of septate uterus includes hysteroscopic resection of the septum (hysteroscopic metroplasty/septoplasty) or abdominal metroplasty procedures. However, limitations on the data regarding the reproductive outcome of women with a uterine septum causes difficulty in making strong recommendations regarding the treatment options (ASRM, 2016).

## Materials and methods

From July 2020 to October 2020, we searched for relevant free full text articles published in PubMed from the 1st of January 2000 to 31st of July 2020. We used text words/keywords such as: congenital anomalies, diagnose, diagnosis, hysteroscopic, hysteroscopy, infertility, metroplasty, miscarriage, pathophysiology, pregnancy, pregnancy loss, prevalence, reproduction, reproductive outcome, septa, septal, septate, septum, treatment, uteri, uterine, uterus. Initially articles that were recognised as duplicates or not written in English were excluded. Additionally, all the potentially eligible papers were independently evaluated by reading the title and abstract. When it was not possible to assess the eligibility of the article by only reading title and abstract, the authors read the full text. After screening the title, abstract and full text, the articles included were systematic reviews, randomised controlled studies, prospective and retrospective cohort studies that investigated the diagnosis, prevalence, pathophysiology and treatment of septate uterus and the reproductive outcome of women with this congenital anomaly. Case-control studies, uncontrolled observational studies and case series were excluded. Authors received no specific funding for this work. This study was a literature review and patients were not involved in setting the research question or the outcome measures, nor were they involved in the design and implementation of the study or dissemination of results.

## Results

**Figure 1:**
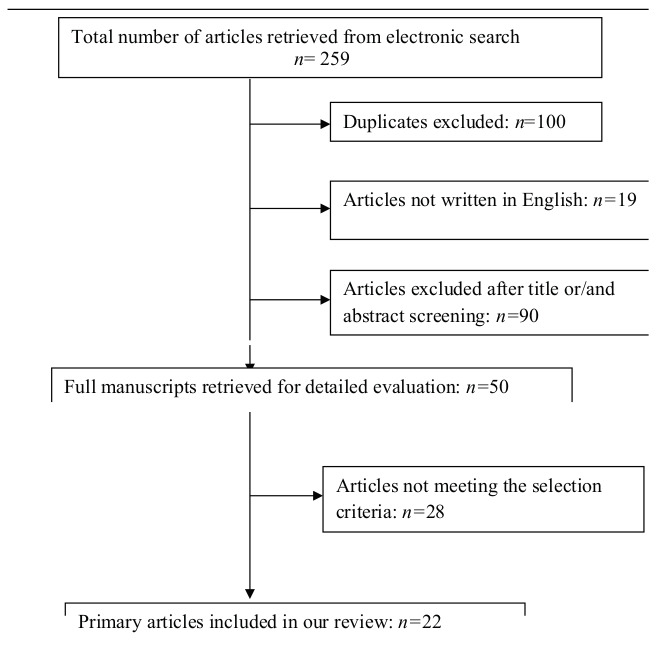
Study selection process

The search yielded 259 articles, all of which were available as free full text in PubMed. 100 of these were excluded as duplicates and 19 of these were excluded as not written in English. Another 90 articles were excluded, as it was clear from the title and abstract that they did not fulfill the selection criteria. We obtained full manuscripts of the remaining 50 articles and, following scrutiny of these, finally identified 22 relevant studies (Figure 1). All twenty-two studies included in the final analysis were systematic reviews, randomised controlled studies, prospective and retrospective cohort studies. All the studies investigated the diagnosis, prevalence, pathophysiology and treatment of septate uterus alongside the reproductive outcome of women with this congenital anomaly.

### Definitions

Three main systems for the classification of septate uterus are in current use: the American Fertility Society’s (AFS) American Society of Reproductive Medicine (ASRM) system; the Congenital Uterine Malformation by Experts (CUME) criteria; and the European Society of Human Reproduction and Embryology (ESHRE) and European Society for Gynaecological Endoscopy (ESGE) consensus on the classification of female genital tract congenital anomalies. According to the ASRM guideline of 2016, septate uterus is defined by the presence of an internal indentation at the fundal midline deeper than 15 mm and by an indentation angle less than 90°. Normal arcuate uterus is defined by an internal indentation less than 1cm in depth with an indentation angle more than 90°. It is clear that these definitions create a grey zone of uncategorised patients, whose uteri contain an internal indentation between 10 mm and 15 mm in depth (ASRM, 2016). On the other hand the ESHRE-ESGE classification proposed a new definition for septate uterus, according to which septate uterus is any uterus with a normal outline and an internal indentation at the fundal midline exceeding 50% of the uterine wall thickness. Additionally, two subtypes of septate uterus were proposed. Partial septate uterus when the septum is dividing partly the uterine cavity above the internal cervical os and complete septate uterus when the uterine cavity is fully divided to the level of the internal cervical os (Grimbizis et al., 2013). As for the CUME classification, septate uterus is defined when the depth of the internal indentation is more than 10mm and the indentation angle is less than 140o or when the indentation-to-wall thickness ratio is greater than 110o (Ludwin et al., 2018). It seems that the agreement between ESHRE-ESGE, ASRM and CUME criteria is poor. The presence of a septate uterus is much more frequent when using the ESHRE-ESGE criteria compared to when using the ASRM criteria (RR, 13.9; 95% Cl, 5.9- 32.7; P < 0.01). In comparison with the CUME criteria, the presence of a septate uterus is significantly higher when using the ESHRE-ESGE criteria (RR, 4.5; 95% Cl, 2.9-6.8; P < 0.01), and significantly lower if using the ASRM criteria (RR, 0.3; 95% Cl, 0.1-0.8; P = 0.01) (Ludwin et al., 2018),

### Embryology, Pathophysiology, prevalence of septate uterus and reproductive outcome

Between the 7^th^ and 9^th^ week of gestation, the fusion of the caudal parts of Müllerian ducts (paramesonephric) occurs and thus there is formation of the common elements of the female genital tract (uterus, cervix, upper two thirds of the vagina). Between the 9th and 20th week of gestation there is absorption of the midline septum and formation of uterine cavity (ASRM, 2016). The processes of fusion and absorption seem to have a continuity and there is overlap, thus an absorption defect might coexist with a fusion defect. Depending on the embryological defect in each patient, the septum’s musculature might have a different consistency (Fascilla et al., 2019). According to a systematic review on the pathophysiology of the intrauterine septum it seems that an intrauterine septum consists of both of endometrial and myometrial tissue and that most of it is vascularised. However, lower number of glandular cells and cilia, incomplete cilliogenesis of the ciliated columnar epithelium cells and lower levels of vascular endothelial growth factor (VEGF) receptors result in a totally different histological composition of the endometrium of the intrauterine septum compared to that of the normal uterine wall (Rikken et al., 2019). A recent publication regarding the consistency of the septum concluded that the septum is a complex structure consisting of islands of muscle fibers, irregularly arranged in a complex of collagen tissue, similar to the structure of myomas (Fascilla et al., 2019). Furthermore, endometrial vascularization of a uterine septum is likely to present alterations which may lead to reduced vascular supply in relation to the rest of the uterus (Salim et al., 2003; Chan et al., 2011a; Rikken et al., 2019). Abnormalities in the vascularisation of the septum’s endometrium and the architecture of the septum’s musculature, seem to have an impact on the later vascular stages of implantation, the receptivity of the uterine septum to the invading trophoblast and on the normal uterine contractility and motility (Salim et al., 2003; Chan et al., 2011a; Venetis et al., 2014; Fascilla et al., 2019). Furthermore, gene expression of the HOAX10 genes, which is known to be important in early embryonic implantation, was found to be altered in women with septate uterus (Rikken et al., 2019). Finally, the length of the septum seems to play an important role in the presence of reproductive problems (Valle and Ekpo, 2013). Recent data suggest that the prevalence of septate uterus in infertile women is 3% (95% CI, 1.3–6.7, P=0.422) and 5.3% in women who suffer from miscarriages (95% Cl, 1.7-16.8, P=0.021); when a history of miscarriages and infertility co-exist its prevalence rises to 15.4% (95% Cl, 12.5-19, P<0.001) (Chan et al., 2011b). Pathophysiology of septate uterus does not seem to underlie any potential detrimental effect on the women’s fertility but it is associated with a higher risk of miscarriage, perinatal complications and poor reproductive outcome (Salim et al, 2003; Chan et., 2011a; Chan et al., 2011b). According to a recent meta-analysis however, the possibility of spontaneous pregnancy in women with septate uterus was significantly decreased (RR 0.86, 95% Cl 0.77 to 0.96; P= 0.009; I2 = 0%). Furthermore, the same meta-analysis concluded that women with septate uterus are at higher risk of first trimester spontaneous abortions (RR 2.65, 95% CI 1.39 to 5.06; heterogeneity: P < 0.00001, I2 = 93%) and second trimester spontaneous abortions (RR 2.81, 95% CI 2.13 to 3.71; heterogeneity: P = 0.33, I2 = 14%) compared with women with normal anatomy. In addition, the presence of septate uterus is associated with statistically increased risk of perinatal complications such as preterm delivery (RR 2.04, 95% CI 0.99 to 4.19), fetal malpresentation (RR 4.35, 95% CI 2.52 to 7.50), intrauterine growth restriction (RR 2.54, 95% CI 1.04 to 6.23), placental abruption (RR 4.37, 95% CI 1.12 to 17.08) and perinatal mortality (RR 2.43, 95% CI 1.10 to 5.36) (Venetis et al., 2014).

### Diagnosis

Screening for septate uterus is an essential part of the clinical investigation of infertile women, women with history of recurrent miscarriages, preterm labour and other perinatal complications. Historically, diagnostic hysteroscopy and laparoscopy have been used as a combined method for accurate diagnosis as they offer direct visualisation of the interior and the exterior of the uterus, and their accuracy has been reported as 100% (Rackow and Arici, 2007; Valle and Ekpo, 2013; ASRM, 2016). More recently, radiological methods are typically used to diagnose the septate uterus due to their significant improvement over the last two decades. In addition, they are less invasive compared to hysteroscopy and laparoscopy. HSG is often the initial test that may provide evidence of a congenital uterine anomaly in patients with poor reproductive outcomes (ASRM, 2016). It is a very accurate method for diagnosing a division of the uterine cavity (Valle and Ekpo, 2013). However, its diagnostic accuracy for septate uterus is low and ranges from 56% to 88% compared with hysteroscopy/laparoscopy. When HSG is combined with ultrasonography its accuracy is estimated to increase up to 90% (Rackow and Arici, 2007). 3D ultrasound is available in everyday gynaecological practice, with high-resolution transvaginal probes, facilitating diagnosis and management of the vast majority of septate uterus cases. Its diagnostic accuracy for septate uterus is 88% compared with hysteroscopy/laparoscopy. Additionally, 3D ultrasound can be combined with saline infusion (saline infusion sonography-SIS) which results in a 100% diagnostic accuracy. MRI is another radiological method used for diagnosis of congenital uterine anomalies. Although there is limited data comparing its diagnostic accuracy for septate uterus with hysteroscopy/laparoscopy, some recent studies suggest that the level of agreement between MRI and other radiologic techniques on the diagnosis of septate uterus is significant (ASRM, 2016). MRI is not frequently used for evaluation of septate uterus due to its high cost, but it is invaluable in complex situations where other methods fail to provide a precise diagnosis (Valle and Ekpo, 2013). A recent randomised controlledtrial (RCT) which examined the impact of modern diagnostic criteria on the reproducibility of the hysteroscopic diagnosis of the septate uterus concluded that hysteroscopy as a single diagnostic tool is insufficient. Hysteroscopy should be combined with other diagnostic techniques such as MRI or 3D ultrasound so that the risk of misclassification of a septate uterus is significantly reduced (Smit et al., 2015).

### Treatment and reproductive outcome after treatment

The abdominal procedures that were mainly performed in the past for resection of the uterine septum include the Jones type and the Bret-Tomkins type metroplasty. These procedures have largely been abandoned because they required laparotomy and hysterotomy, exposing patients to the formation of postoperative adhesions, in addition to prolonged recovery, 3-6 months waiting time before attempting conception and mandatory caesarean section as a route of delivery in subsequent pregnancies (Valle and Ekpo, 2013; ASRM, 2016). On the other hand, the hysteroscopic approach for the management of uterine septa has prevailed in recent times as it significantly reduces the adverse effects and morbidity associated with the procedure. Mechanical scissors; electrosurgery with specially designed electrodes fitted to the hysteroscope or resectoscope; vaporising or bipolar electrodes; lasers of the fiber type and the argon laser with extruded tips; and mechanical morcellators can be used to accomplish hysteroscopic septoplasty. Hysteroscopic scissors and the resectoscope fitted with an appropriately designed electrode are the most commonly used instruments (Valle and Ekpo, 2013). A recent meta-analysis looked at perinatal outcomes between the use of monopolar resectoscope when compared with blunt scissor for the treatment of congenital uterine septum. This study analysed differences in fertility indices between diathermy and scissors. No significant difference was found between groups for the primary (live birth rate and pregnancy rate) or the secondary perinatal outcomes (term birth, preterm birth, miscarriage rates). The pregnancy rate for scissors was 88.8% and for resectoscope was 75.6% (OR: 2.13, 95% CI: 0.65-6.98, I2=29%; P=0.23). Delivery rates were 78.1% for scissors and 75% for resectoscope (OR: 1.29, 95% CI: 0.46- 3.60, I2=0%; P=0.53). Miscarriage rates were 21.8% for scissors and 27.1% for resec¬toscope (OR: 0.78, 95% CI: 0.28-2.17, I2=0%; P=0.53). Preterm delivery rates were 6.2% for scissors and 6.7% for resectoscope (OR: 0.85, 95% CI: 0.16-4.42, I2=0%; P=0.94). Term deliv¬ery rates were 71.8% for scissors and 66.1% for resectoscopic treatment (OR: 1.32, 95% CI: 0.53- 3.57, I2=0%; P=0.47). The results indicate that the ef-ficacy regarding perinatal outcome is similar between the two different hysteroscopic techniques (Daniilidis et al., 2020). Hysteroscopic metroplasty can also be combined with laparoscopic or sonographic guidance in order to diagnose pelvic pathology, assess tubal patency and prevent uterine perforation, with results being similar regarding safety and efficacy (Nouri et al., 2010; ASRM, 2016; Rikken et al., 2018). According to recent studies that evaluated the reproductive outcomes after hysteroscopic treatment of uterine septum, pregnancy rates range from 60.1% to 67.5% and live birth rates range from 45% to 54.7% (Nouri et al., 2010; Bandifallah et al., 2013; Valle and Ekpo, 2013). However, it is still unclear if there is a significant difference in the reproductive outcome between women who underwent hysteroscopic excision of the uterine septum and those who chose expectant management (Rikken et al., 2017; Rikken et al., 2018; Rikken et al, 2020). Hysteroscopic metroplasty is indicated in women with recurrent miscarriages as it significantly improves their reproductive outcome. It is associated with good pregnancy rates (80% - 90%) and good live birth rates (53.7%). However, its role on the treatment of women with primary infertility still remains controversial. Recent studies reported a significantly lower number of pregnancies in those women after hysteroscopic metroplasty (40% - 53.1% pregnancy rate), with live birth rates being comparable due to the small number of miscarriages after treatment ( Homer et al., 2000; Pabuccu and Gomel, 2004; Rackow and Arici, 2007; Bosteels et al., 2009; Bendifallah et al., 2013; Valle and Ekpo, 2013) Although hysteroscopy before an in-vitro fertilisation (IVF) attempt has not been proven to favour higher pregnancy rates in women with history of recurrent IVF failure, some recent studies concluded that hysteroscopic septoplasty seems to be beneficial for infertile women with septate uterus who plan to undergo IVF, and especially for those without any other known cause of infertility (Rackow and Arici, 2007; Mollo et al., 2008; Bendifallah et al., 2013; El- Toukhy et al., 2016).

## Discussion

The presence of septate uterus is significantly different when using the three main systems of classification: the ASRM system, the CUME criteria and the ESHRE-ESGE system. Lack of uniformity within these classification systems means that the differential diagnoses of septate and arcuate uterus are still not universally accepted and highlights the need for worldwide consensus on the diagnostic criteria of septate uterus (Grimbizis et al., 2013; Venetis et al., 2014; ASRM, 2016; Ludwin et al., 2018). The above fact also has an impact on the assessment of the different diagnostic methods, regarding their accuracy on the diagnosis of septate uterus, and on the evaluation of prevalence of septate uterus. Some of the most-commonly used methods among the last decades such as two-dimensional ultrasound, HSG and hysteroscopy are now considered suboptimal. Additionally, laparoscopy combined with hysteroscopy, despite being accurate methods, are now called into question as they are invasive procedures. On the other hand, many recent papers provide fair evidence that 3D ultrasound, MRI, sonohysterography and combination of imaging with hysteroscopy are more optimal methods due to their accuracy and the advantage of being less invasive. Unfortunately, there is still no method considered to be the gold standard for accurate diagnosis (Chan et al., 2011b; ASRM, 2016). Although the majority of women with septate uterus do not face reproductive difficulties, there are sufficient data from recent papers associating the presence of the intrauterine septum with reduced conception rate, miscarriage, preterm labour and increased risk of adverse pregnancy outcomes such as malpresentation, intrauterine growth restriction, placental abruption and perinatal mortality (Salim et al., 2003; Rackow and Arici, 2007; Chan et al., 2011a; Venetis et al., 2014; ASRM, 2016). On the other hand, there is still not sufficient evidence in the recent literature associating the presence of septate uterus and its pathophysiology with infertility (Chan et al., 2011a; Chan et al., 2011b; ASRM, 2016). However, according to new insights into the pathophysiology of the uterine septum it seems that not all septa are the same, and this could be the expression of differences in the underlying embryological defects that led to their formation. Failed reabsorption of the midline septum is the main and obvious defect in women with septate uterus, but an incomplete and obscure fusion might also coexist. This seems to be associated with differences in the histological composition and vascularisation between septa as well as in clinical significance. We could hypothesise that a ‘pure’ reabsorption defect with normal fusion might be associated with a ‘fibrous’ and less vascularised septum, accompanied by changes in the overlying endometrium. These women might experience subfertility. On the other hand, a ‘mixed’ reabsorption and obscure fusion defect might be associated with a more ‘muscular’ and vascularised septum leading to an abnormal pattern of muscular architecture and uterine motility. These women might experience recurrent pregnancy loss and preterm delivery. The current hypothesis for the septa is based on the fact that the main histological background of septa is the alterations in the myometrial consistency at the level of the fundus, and this could lead to alterations in vascularisation, alterations in the overlying endometrium, alteration of expression of hoax genes related to implantation, impairment of embryo adherence and pregnancy evolution. On the other hand, alterations in the myometrial consistency could lead to abnormal motility patterns contributing to the impairment of embryo adherence (Fascilla et al., 2019; Rikken et al, 2019). Data regarding the beneficial effect of septate uterus’ hysteroscopic treatment on reproductive outcome are still limited on the literature. The small number of studies indicating the association of hysteroscopic treatment with reduction of miscarriage rates and improvement in live birth rates in women with history of recurrent abortion or infertility, strengthens a continuous dilemma whether hysteroscopic treatment or expectant management should be suggested in these patients (ASRM, 2016; Rikken et al., 2017., Rikken et al., 2018; Rikken et al., 2020). Additionally, there are limited studies and no systematic reviews comparing the different hysteroscopic techniques (resectoscope, scissors, laser) with regard to reproductive outcome. A recent meta-analysis comparing monopolar resectoscope with blunt scissors concluded that the efficacy regarding perinatal outcome was similar between the two techniques. Therefore, the standard criterion for choosing the appropriate treatment method for septate uterus should be based upon the opera¬tor’s experience and not upon the hysteroscopic instrument itself. Nevertheless, further evidence is required and additional RCTs are needed to accurately determine the efficacy of hysteroscopic procedures, rendering a definitive recommendation premature at this point (Daniilidis et al., 2020).

## Conclusions

Septate uterus is associated with poor reproductive outcome. Failed reabsorption of the midline septum is the main and obvious defect in women with septate uterus, but an incomplete and obscure fusion might also coexist leading to differences in the histological composition and vascularisation between septa as well as in clinical significance. Alterations in vascularisation and motility pattern might result in impaired implantation and pregnancy outcome. Lack of uniformity with the classification systems of septate uterus makes the diagnosis of septate challenging. More RCTs are needed to provide strong data on the accuracy of diagnostic methods, on the pathophysiology and prevalence of septate uterus and on the reproductive outcome of patients. Finally, well-designed research with validated data regarding the optimal strategy for treatment of the septate uterus is needed in order to minimise bias.
